# Pre-hospital Hemorrhagic Control Effectiveness of Axiostat® Dressing Versus Conventional Method in Acute Hemorrhage Due to Trauma

**DOI:** 10.7759/cureus.5527

**Published:** 2019-08-29

**Authors:** Mohamed Kabeer, P. P Venugopalan, V. C Subhash

**Affiliations:** 1 Accident and Emergency Medicine, Hamad General Hospital, Doha, QAT; 2 Emergency Medicine, Aster Malabar Institute of Medical Sciences, Ltd., Kozhikode, IND; 3 Surgery, Aster Malabar Institute of Medical Sciences, Ltd., Kozhikode, IND

**Keywords:** bleeding, chitosan, cotton, dressing, trauma, wound, axiostat

## Abstract

Accidents and trauma are one of the leading causes of death and disability throughout the world. In developing countries like India where emergency trauma care is still emerging, it accounts for almost 10% of deaths every year. Lack of adequate pre-hospital care and uncontrolled bleeding from the wound site are stated to be the prominent reasons for such deaths. The aim of this study was to evaluate the efficacy of a novel chitosan-based haemostatic dressing, Axiostat® (Axio Biosolutions Private Ltd., Gujarat, India), as a hemorrhage control device in the ambulance setting.

A total of 104 patients with bleeding scalp wounds were randomly allocated into two treatment groups while transporting them to the hospital. Patients in Group I were treated with Axiostat® chitosan haemostatic dressing (n = 47), while a conventional cotton gauze dressing was used in Group II (n = 57). A standard procedure was followed to apply the dressing on bleeding wounds and time to achieve haemostasis, the amount of blood loss, the number of patients with haemostasis, the occurrence of rebleeding, and other side effects were noted.

The mean age of the patients was 40 years and the majority of patients were male - 73 (70%). Most of the wounds were lacerations with venous bleeding. Haemostasis time was 4.68 ± 1.04 minutes and 18.56 ± 5.04 minutes in the Axiostat® and cotton gauze groups, respectively. The use of Axiostat® significantly reduced the time to haemostasis (p < 0.0001). A significant reduction in blood loss was observed with the application of Axiostat®. Successful haemostasis was achieved in 94% of patients in the Axiostat® group and 74% patients in cotton gauze group, respectively (p < 0.05). Moreover, no side effects, such as tissue loss or rebleeding at time of removal, were seen with the use of Axiostat®, while three patients in the cotton gauze group showed some side effects.

Results show that Axiostat® enables rapid haemostasis and can prevent significant blood loss during emergency trauma and accidents. Additionally, it also allows for easier removal from the wound site without leaving any residue, which helps in rendering the wound clean. In conclusion, the study successfully demonstrates the potential of Axiostat® as a first-line intervention in controlling acute haemorrhage in emergency care.

## Introduction

According to the World Health Organization (WHO), road traffic accidents are one of the major causes of traumatic injuries leading to death worldwide. The literature states that about one in 10 deaths of the total mortality (that is more than 5.8 million deaths per year) happen due to traumatic injuries [1]. If we throw light on the Indian scenario, the surprising fact is that more Indian people have lost their lives on the roads than any natural disaster or terrorist attacks [2]. As per the National Crime Records Bureau (NCRB) report, a total of 464,674 cases of road accidents were observed in 2015, which is a 3.1% increase from the previous year, and out of that, 148,707 cases led to a fatality [3].

A recent study suggests that 82% of the deaths occur because of uncontrolled bleeding out of total preventable combat deaths [4]. Moreover, mortality due to excessive bleeding contributes to about one-third of the total traumatic deaths in the world [5]. Laceration or fracture of the scalp contributes to 60% of the total traumatic haemorrhage incidences [6]. Uncontrolled bleeding compromises the balance between the demand and supply of the tissue oxygen at the injury site which can ultimately lead to cardiac arrest [7]. If efforts are made to stop bleeding, death would be preventable. Conventionally, cotton gauze is used with pressure to stop bleeding as the first line of treatment, which may not be always successful and has many limitations. The ideal characteristics of the haemostatic dressing include the ability to stop bleeding quickly, absorb blood, simple application and removal, and it should be porous enough to allow oxygen transfer to the wound site [8-9]. Apart from these properties, a haemostatic dressing should have antimicrobial properties and be non-allergic in nature as it is directly exposed to the open bleeding wound [9]. Today’s need is a topical, simple, and advanced haemostat to stop bleeding rapidly before the patient reaches the hospital [10]. In recent years, several haemostatic agents, effective through a different mechanism of actions, have been developed and studied to efficiently control severe bleeding as compared to conventional cotton gauze [11].

Recent literature explains the potential and use of chitosan dressings as a haemostat [12]. Chitosan has proved to be an effective haemostatic agent so far and has a range of applications in wound healing because of its properties [13-14]. It is a natural biomaterial and deacetylated form of chitin. Chitosan is mainly composed of glucosamine and N-acetyl glucosamine residues with a 1, 4-β-linkage. It is polycationic in nature; the presence of primary amines (-NH2) in chitosan gives it a net positive charge. It is antimicrobial and has other important properties, such as biocompatibility and biodegradability.

The objective of this study was to evaluate the safety and efficacy of the chitosan dressing, Axiostat® (Axio Biosolutions Private Ltd., Gujarat, India), in comparison with conventional cotton gauze as a pre-hospital dressing to stop bleeding from scalp wounds.

## Materials and methods

This prospective, open-label study was carried out between May 2012 and June 2013 at the Department of Emergency Medicine, Malabar Institute of Medical Science, Kerala, India. The study protocol was approved by the institutional ethics committee and written consent was taken from all patients/patient’s legal representative/impartial witness for participation in the study.

Patients included in the study were greater than or equal to 18 years old with bleeding wounds over the scalp. For inclusion in the study, wounds must be bleeding at the time of baseline assessment and wound size should be covered by a single available size of study device. Patients who had a prior diagnosis of disease or medical condition affecting the ability of blood to clot (e.g., hemophilia), a non-survivable injury as per the investigator’s discretion, patients who, in the opinion of the investigator, may not complete the study for any reason (e.g., patients requiring immediate suturing), grossly infected wounds which may require multiple debridement procedures prior to clearance of bacteria, and non-viable tissue from the wound were excluded from the study. Patients currently participating in an investigational drug or device study that had not yet completed its primary endpoint or interfered with procedure and assessments in this study, patients with a surgical/iatrogenic wound, and patients with a major head injury, spinal injury, neck injury, abdominal injury, deep wound injury, fracture, haemorrhagic shock, or foreign materials inside the wound, such as a stab injury, were also excluded from the study. The size of the dressing was 8 cm x 5 cm in both groups.

**Figure 1:**
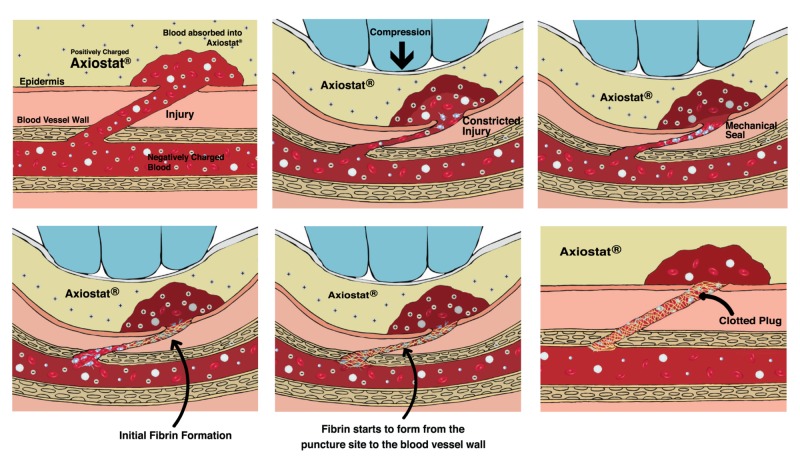
Mechanism of action of Axiostat®

Axiostat® is a sterile, single-use, 100% chitosan dressing designed to stop bleeding instantly. The positively charged chitosan in Axiostat® binds to negatively charged blood components and exhibits bio-adhesiveness. The dressing mainly works on the principle of electrostatic interaction which bypasses the natural clotting mechanism to create a strong mechanical seal (Figure 1). The highly porous structure of Axiostat® enables the rapid absorption of plasma from the blood, which leads to the accumulation of erythrocytes and platelets at the injury site. Furthermore, Axiostat® activates the platelets and triggers the blood coagulation pathway. This pathway eventually results in fibrin formation and creates a plug extending throughout the site of injury, thereby stopping the bleeding. The wound remains closed due to the fibrin mesh. The dressing is pH-responsive and can be easily removed using saline. In Group II, conventional cotton gauze was used.

Patients were recognized and enrolled in the study by emergency medical care technicians (EMCT). These technicians underwent a one-year EMCT course under the ANGELS (Active Network Group of Emergency Life Savers) ambulance network in Kerala and were trained for the study protocol. After each use of the dressing, the technicians recorded data on a case report form.

Ambulances were equipped with Axiostat® chitosan haemostatic dressing, along with conventional cotton gauze, for the duration of this study. Axiostat® or the conventional cotton gauze dressing was used as an emergency haemostat on scalp wounds while transporting trauma patients to the hospital. Both the dressings were applied to the bleeding wound with the same predefined protocol. Moderate pressure was applied by the fingers for about two minutes until the dressing adhered to the bleeding wound site and stayed in the position. The time at which bleeding ceased was noted as a time to achieve hemostasis and was recorded as a primary efficacy endpoint. If the bleeding was persistent, a second application of the respective dressing was applied. Upon failure to achieve haemostasis after two applications, patients were treated as per the institutional standard of care. The secondary endpoint was to record the amount of blood loss, number of patients requiring a second application, number of patients with haemostasis, and side effects, like the difficulty in removal of the dressing due to adherence, tissue loss, rebleeding, and allergy. In both groups, the dressing was kept for 30 minutes after haemostasis was achieved. Axiostat® was removed by applying saline and gently lifting it off. Cotton gauze was removed as per the institutional standard care practice. After the removal of the dressing, the patients were further treated as per the institutional standard of care.

Sample size calculation and statistical analysis

The clinical effect of interest was the time to achieve the haemostasis, compared to the control group, of 12 minutes. The expected standard deviation in the population in which the study was undertaken was 20. Thus, the standard difference equates to 12/20 = 0.6. With the Type I error of 5% and study power of 80%, the minimum number of patients to be allocated in each group was determined to be 45. Thus, the sample size of 45 subjects in each arm of the study and a total sample size of at least 90 subjects was sufficient to prove the objective. The time for hemostasis and amount of blood loss were expressed as mean ± standard deviation (SD) and analyzed with Student’s t-test. Categorical variables (age, gender, type of injury, and location of the injury site) were expressed as absolute frequencies and percentages and analysed as applicable. The analysis was performed using the Statistical Package for Social Sciences (SPSS) (IBM SPSS Statistics, Armonk, NY), version 21.

## Results

A total of 133 patients with bleeding scalp wounds were identified for this study out of which 29 patients were excluded because they did not meet the study criteria (incomplete data collection, injuries other than scalp injuries, etc.). Out of the remaining 104 patients included in the study, 47 (45.2%) patients were treated with Axiostat® (Group I) and 57 patients (54.8%) with cotton gauze (Group II). The mean age of the patients from Group I was 42 and consisted of 32 males and 15 females. Similarly, the mean age of the patients of Group II was 40 and consisted of 41 males and 16 females. The pre-test parameters, including the type of wound, duration between injury and dressing application, and the location of the wound, were recorded. There was no significant difference between the pre-test parameters (Table 1).

**Table 1:** Comparison of the Pre-test Parameters of Both Groups SD: standard deviation

Parameters	Axiostat®	Cotton Gauze	P-value	Test
Gender			0.67	Chi 2
Male	32 (68%)	41 (72%)		
Female	15 (32%)	16 (28%)		
Age, mean ± SD	42.19 ± 11.72	40.05 ± 12.78	0.41	Mann-Whitney
Wound duration			0.15	Chi 2
< 1 hours	46 (97.87%)	52 (91.23%)		
1 - 3 hours	1 (2.13%)	5 (8.77%)		
Type of wound			0.26	Chi 2
Lacerations, venous bleeding	13 (27.66%)	12 (21.05%)		
Lacerations, venous bleeding, trauma, others (arterial bleeding)	0 (0.00)	4 (7.02%)		
Lacerations, venous bleeding, trauma	30 (63.83%)	32 (56.14%)		
Puncture wound, trauma, others (arterial bleeding)	0 (0.00)	1 (1.75%)		
Puncture wound, venous bleeding, trauma	3 (6.38%)	4 (7.02%)		
Puncture wound, venous bleeding, trauma, others (arterial bleeding)	0 (0.00)	2 (3.51%)		
Trauma, others (arterial bleeding)	1 (2.13%)	2 (3.51%)		
Location of wound			0.45	Chi 2
Forehead	8 (12.02%)	7 (12.28%)		
Left parietal area	6 (12.77%)	9 (15.79%)		
Right frontal area	12 (25.53%)	9 (15.79%)		
Right parietal area	11 (23.40%)	11 (23.40%)		
Other scalp wounds	10 (21.27%)	21 (36.84%)		

**Figure 2:**
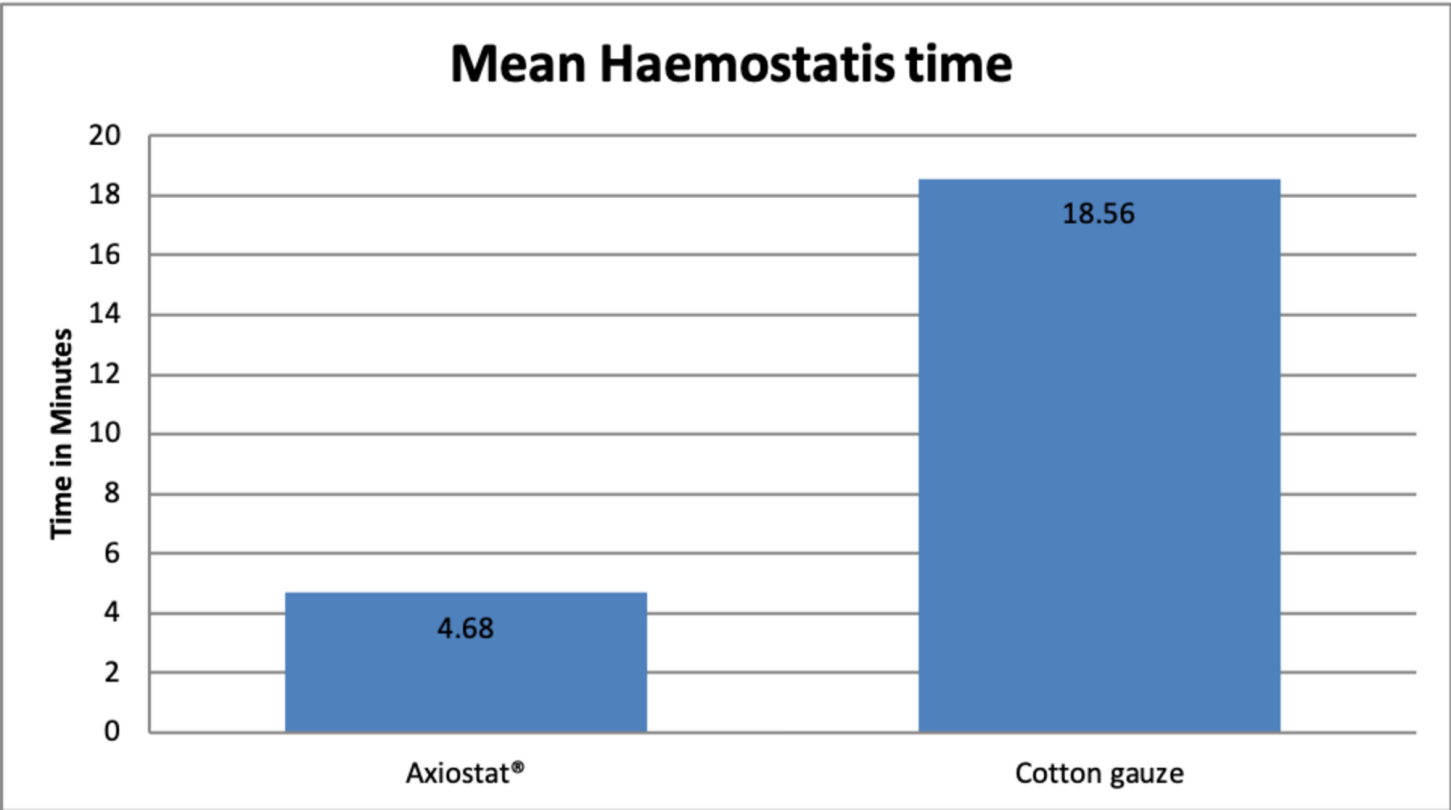
Haemostasis time with the use of Axiostat® and cotton gauze

The average time taken to achieve haemostasis in the Axiostat® group (Group 1) was 4.68 ± 1.04 minutes, which is a significantly lower p-value < 0.0001 than that in cotton gauze group (Group II) which was 18.56 ± 5.04 minutes. Results demonstrate faster haemostasis in the Axiostat® group (Figure 2).

**Figure 3:**
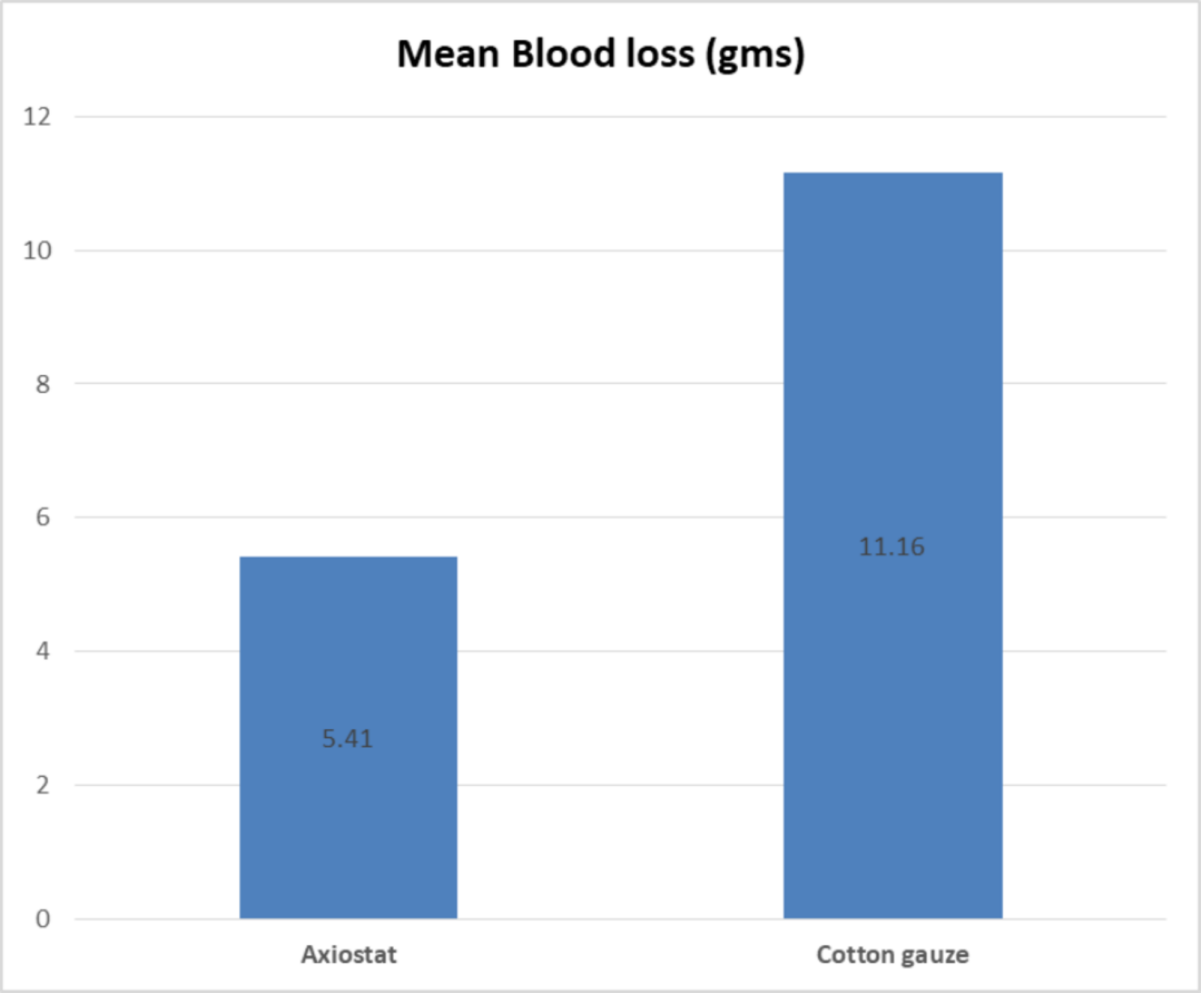
Blood loss with the use of Axiostat® and cotton gauze

Blood loss was evaluated by measuring the weight of the dressing after the first application. Since each material was supplied in a sterile pack and weighing each sample was not possible, the average weights of both materials were taken before application, which were 13.70 g and 4.80 g for cotton gauze and Axiostat®, respectively. Blood loss through the injury site was observed as 11.16 ± 4.96 g for cotton gauze, which is significantly higher (p < 0.0001) than that in the Axiostat® group 5.41 ± 2.53 g (Figure 3).

Twenty (35%) patients in the cotton gauze group required two dressing applications, whereas, in the Axiostat® group, only eight (17%) patients required a second application of the dressing. This result was significant at p < 0.05 (Table 2.) In the Axiostat® group, successful haemostasis was achieved in 44 (94%) subjects as compared to 42 (74%) subjects in the cotton gauze group. This result was found to be statistically significant at p < 0.05.

**Table 2:** Number of Dressing Applications and Rebleeding

Parameters	Axiostat® freq (%) (N = 47)	Cotton Gauze freq (%) (N = 57)	P-value (Chi-square test)
Number of patients with two dressing applications	8 (17%)	20 (35%)	.038712 (p < 0.05)
Number of patients with haemostasis	44 (94%)	42 (74%)	.007492 (p < 0.05)

Side effects with the use of the dressings were evaluated and included the difficulty in removing the dressing for suturing due to adherence, tissue loss, and rebleeding on removal. No side effects were observed for patients treated with the Axiostat® dressing, whereas three patients in the cotton gauze group showed some side effects. Allergic reactions with the use of the dressing were studied which included mild itching, redness, and mild local swelling. More reactions were observed with the cotton gauze (i.e., four patients) and only one patient reported signs of mild allergy with Axiostat®. No severe allergies or adverse events were observed in any case. All subjects needed suturing as those included in the study had a scalp wound potential for rebleeding.

## Discussion

In the last few decades, substantial research has been done on haemostatic dressings. Over the years, many types of dressings have been used to stop bleeding which include conventional cotton gauze, as well as interactive dressings like polyurethane membrane and foam [15-16]. The most widely used cotton gauze dressings can cause pain at the time of removal. Furthermore, if the size of the wound is large, the time for haemostasis would be prolonged. This can be fatal if the patient is not taken in for medical consideration on time. Nowadays, many researchers are inclined towards advanced techniques for haemorrhage control using bioactive material as a haemostatic dressing, such as collagen, chitosan, alginate, gelatin, etc. [17-19].

Chitosan dressings are found to be safe and effective to treat prehospital haemorrhage [20-22]. Chitosan has a positive charge because of its amino functional group which causes an electrostatic interaction with the negatively charged blood cell membrane that ultimately leads to the clot formation [23]. Since Axiostat® is made up of 100% chitosan, it shows the most efficacious haemostatic activity irrespective of any blood coagulation disease and bypasses the natural blood-clotting mechanism [14, 24].

Haemostasis is the first property of a dressing intended to be used to control bleeding. In this clinical study, haemostasis was achieved in 83% of the patients with the first application of Axiostat®. Time to achieve haemostasis observed with the use of Axiostat® was significantly lower than the conventional cotton gauze group. Moreover, there was about a 50% decrease in blood loss from the wound site in cases of Axiostat®-treated patients compared to the control group. Axiostat® was found to be safe as no rebleeding, tissue loss, or adverse reactions were observed with the use of this dressing.

There are many haemostatic dressings available for pre-hospital bleeding management which include kaolin-based, zeolite-based, and chitosan-based haemostats, such as QuikClot® (Z-Medica LLC, Wallingford, CT), Celox™ (Medtrade Products Ltd., Crewe, UK), ChitoFlex® (HemCon Inc., Portland, OR), ChitoGauze® (Tricol Biomedical, Inc., Portland, OR), etc. [25]. Chitosan-based haemostatic dressings are effective in controlling both arterial and venous haemorrhage [9, 26]. The same are effectively used for moderate to severe bleeding and also used in patients with coagulopathy [26-27]. Furthermore, these types of dressings are mainly intended to be used for laceration, trauma, and combat settings, such as bleeding gunshot wounds [17, 28]. While comparing results of this study with similar advanced chitosan haemostatic dressings, the time to achieve haemostasis was found to be significantly lesser as compared to ChitoGuaze dressing and it remains approximately the same with Celox and ChitoFlex bandages. Chitosan dressings have been successfully used in civilian stab wounds, civilian emergency medical services, and femoral artery swine model bleeding [21, 26, 29]. Considering safety parameters, chitosan dressings are found to be safe and without any significant adverse effects [22, 30].

There were some limitations to this study. It does not include the prior history of patients and the overall population of this study was small; however, it allows statistical analysis. Data was collected by various emergency medical technicians; some of the data collected were incomplete so those patients had to be excluded from the study. Besides that, only scalp injuries were selected for this study. It was difficult to design a blinded study as Axiostat® is composed of chitosan and looks different from the standard cotton gauze. In the study, Axiostat® was removed with saline which may affect the weight of dressing for calculation of blood loss. Also, any blood truly shed onto the ground at the time of injury has not been accounted for. Moreover, this study was not designed to record any follow-up data that does not allow us to observe the role of dressing on wound healing. However, the study portrays the efficacy of Axiostat® as an effective haemostatic dressing.

## Conclusions

Within the scope of this clinical study (which included trauma cases with bleeding scalp injuries), it was observed that the chitosan dressing, Axiostat®, significantly reduced time to haemostasis and reduced blood loss during emergency and trauma as compared to conventional cotton gauze. Additionally, it is easy to apply and shows negligible side effects. The study demonstrates that Axiostat® can be used as a simple and effective haemostat in pre-hospital health care situations, such as an ambulance.
